# Development and validation of a clinical cure marker based on negative lymph nodes for gastric cancer after gastrectomy

**DOI:** 10.3389/fsurg.2023.1016252

**Published:** 2023-05-09

**Authors:** Jiebin Xie, Yuan Zhang, Ming He, Xu Liu, Jing Dong, Pan Wang, Yueshan Pang

**Affiliations:** ^1^ Department of Gastrointestinal Surgery, Affiliated Hospital of North Sichuan Medical College, Nanchong, China; ^2^ Department of Geriatrics, Central Hospital of Nanchong, The Second Clinical School of North Sichuan Medical College, Nanchong, China

**Keywords:** gastric cancer, prognosis, negative lymph node, diagnostic markers, negative lymph node/T stage

## Abstract

**Objective:**

To explore lymph node (LN)-related derived indicators as clinical cure markers for gastric cancer (GC) after gastrectomy.

**Methods:**

Data of resected GC patients were extracted from the SEER database and our own department. Propensity score matching (PSM) was used to balance the baseline differences between the clinical cure and the nonclinical cure groups. The area under the curve (AUC) and decision curve analysis (DCA) were used to choose the optimal marker, and survival analysis was used to validate the clinical value of the most effective marker.

**Results:**

After PSM, the differences in age, sex, race, location, surgical type, and histologic type between the two groups were significantly reduced (all P > 0.05), and the AUCs of examined LNs (ELNs), negative LNs (NLNs), ESR (ELNs/tumor size), ETR (ELNs/T-stage), NSR (NLNs/tumor size), NTR (NLNs/T-stage), EPR (ELNs/PLNs) and NPR (NLNs/PLNs) were 0.522, 0.625, 0.622, 0.692, 0.706, 0.751, 7.43, and 7.50, respectively. When NTR was 5.9, the Youden index of 0.378 was the highest. The sensitivity and specificity were 67.5% and 70.3% in the training group and 66.79% and 67.8% in the validation group, respectively. DCA showed that NTR had the largest net clinical benefit, and patients with NTR greater than 5.9 had significantly prolonged overall survival in our own cohort.

**Conclusion:**

NLNs, NTR, NSR, ESR, ETR, NPR and EPR can be used as clinical cure markers. However, NTR was the most effective, and the best cutoff value was 5.9.

## 1. Introduction

Gastric cancer (GC) is the fifth most common cancer and the fourth leading cause of cancer mortality and has become a major health problem worldwide. Annually, an estimated 1.08 million new cases are diagnosed worldwide (1). Currently, surgery is still the main treatment for resectable GC (2). However, only the patients who lived more than 5 years after surgery showed that they had been clinically cured, and the number of lymph node (LN) metastases was the main factor contributing to the 5-year survival rate of GC (3). Therefore, the complete dissection of potential metastatic LN is the basic requirement of radical gastrectomy. However, it is very difficult to precisely judge potential metastatic LNs before and during surgery. In addition, due to the differences in surgeons' awareness, experience, and the technique of D2 LN dissection procedures (4), as well as the differences in patients' factors (5, 6), different clinical outcomes were presented for the same level of radical surgery. Therefore, accurately and objectively evaluating these differences and based on these differences to predict whether GC patients will be clinically cured is difficult. At present, the number and status of LNs in each group are not accurately routinely reported in postoperative pathological reports. The surgical quality was roughly evaluated by examining LNs (ELNs), negative LNs (NLNs), the status and distances of the resection margin (7, 8), but the accuracy needs to be further improved. Whether ELNs and NLNs can predict clinical cure outcomes is unclear.

Using tumor severity indicators such as CEA, T-stage and tumor size to adjust some markers is a common method to improve the prognostic value (9–11). Our recent research revealed that the prognostic value of NLNs was significantly improved by adjusting the tumor size of rectal cancer (10) and T-stage of GC (9). Therefore, to unveil whether the adjusted LN-related derived indicators can be used as predictive clinical cure markers and to screen the most efficient predictive markers, we first retrospectively analyzed the GC data in the SEER database and our department by using receiver operating characteristic (ROC) curves and decision curve analysis (DCA) for further study.

## 2. Materials and methods

### 2.1. Patients

**Figure 1:**
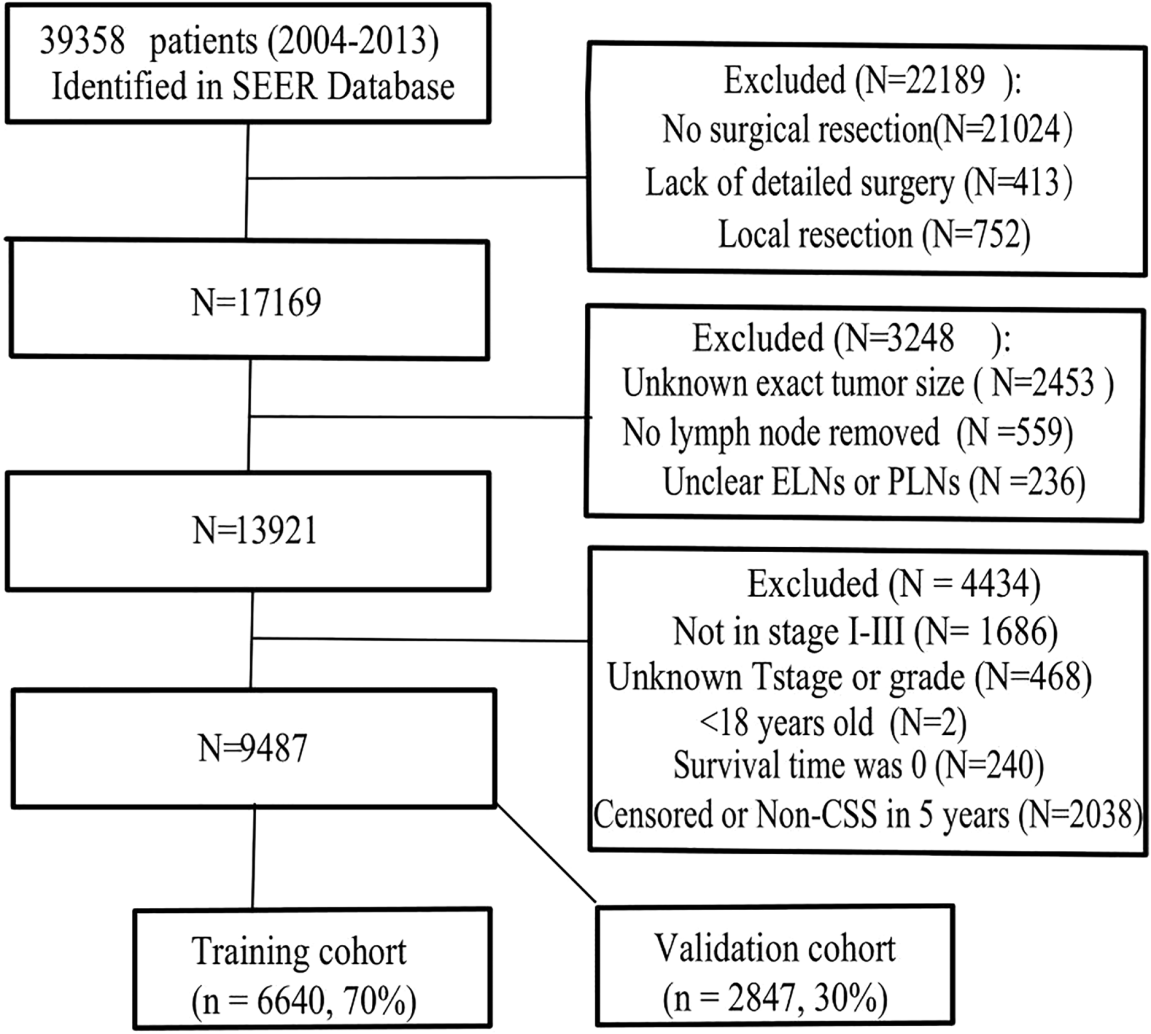
Flowchart of patient selection. ELNs, examining lymph nodes (ELNs); PLNs, positive lymph nodes; non-CSS, cancer-specific survival.

The SEER database is a public tumor database, covering 27.8% of the population in the United States. We obtained permission to obtain research data from the SEER database before the study (Reference Number 11112-Nov2019). We used the same code as in our previous research to extract the clinical information of all GC patients by SEER*stat8.3.8 software (9). All included patients were required to have one primary malignancy who was pathologically confirmed and required a follow-up time greater than 5 years. Finally, we identified a total of 39,358 patients from 2004 to 2013 (Figure 1).

The exclusion criteria were as follows: ① The patient did not receive surgical resection or lacked a detailed description of the surgery. ② Age less than 18 years ③ distant metastasis ④ patients with unknown exact tumor size, unknown tumor differentiation, unclear ELNs or PLNs ⑤ survival time was 0 or noncancer-related death within 5 years. The finally enrolled patients were randomly divided into the training cohort and the validation cohort (7:3) for cross-validation.

We further retrospectively reviewed 510 clinical stage I-III GC patients who underwent D2 gastrectomy without neoadjuvant therapy at our department from June 2015 to May 2019 (Supplementary Figure S1). All included patients were required to undergo laparoscopic or open surgeries performed by experienced associate professors or professors. The final diagnosis and stage were established by histologic examination of the resected specimen according to the 8th edition TNM staging system. The study was approved by the Ethics Committee of the Affiliated Hospital of North Sichuan Medical College (2021ER0102-1) and met the ethical standards set by the Declaration of Helsinki.

### 2.2. Statistical analysis

In the present study, the patients who lived more than 5 years represented clinical cure were assigned 1, and the patients who died of cancer-related diseases within 5 years after surgery represented nonclinical cure were assigned 0. According to the relative hazard ratio of T stage (9), the T1, T2, T3, T4a and T4b stages were, respectively assigned 0.5, 1, 2.5, 4 and 5 to quantify the severity of GC. The positive LN (PLNs) and the tumor size represented the severity by their own number. The ELNs, NLNs, ETR (ELNs/T-stage), ESR (ELNs/tumor size), NTR (NLNs/T-stage), NSR (NLNs/tumor size), EPR (ELNs/PLNs) and NPR (NLNs/PLNs) were analyzed as continuous variables. The chi-square or T test was used to compare the differences in baseline characteristics between the two groups. Propensity score matching (PSM) with a caliper value of 0.005 was used to balance the baseline differences between the clinical cure and the nonclinical cure groups. The area under the ROC curve (AUC) for each marker was calculated, and the clinical benefit was analyzed using DCA. The optimal cutoff value was determined according to its Youden value. The specificity and sensitivity were calculated in the validation and training groups. The Kaplan–Meier method with the log-rank test was used for survival analysis by SPSS 22.0 (Chicago, IL, USA). Other analyses were performed by using R software (v3.6.3, http://www.R-project.org).

## 3. Results

### 3.1. General conditions

A total of 9,487 patients were ultimately included and randomly divided into training (6,640 cases) and validation cohorts (2,847 cases), and no significant difference was observed between the two cohorts (all *P* > 0.05, Table 1). Among the included patients, 5,989 cases were male, 70.3% were older than 60 years, proximal gastric cancer had the highest proportion (39.3%), and most were stage III (42.6%). Only 2.3% of the cases were mucinous adenocarcinoma, and 4,463 (47.1%) cases met the clinical cure criteria. Detailed data of the PLNs, ELNs, NLNs, ESR, ETR, NSR, NTR, and EPR are shown in Table 1.

**Table 1:** Clinicopathological features of patients with gastric cancer*.

Characteristic	All (*N* = 9,487)	Training cohorts (*N* = 6,640)	validation cohorts (*N* = 2,847)	*P*
	cases (%)	cases (%)	cases(%)	
Tumor size	48.20 ± 43.00	48.45 ± 44.36	47.64 ± 39.67	0.4
PLNs	4.07 ± 6.45	4.10 ± 6.46	4.02 ± 6.45	0.575
ELNs	17.42 ± 12.24	17.45 ± 12.25	17.36 ± 12.23	0.739
ESR	0.61 ± 1.10	0.61 ± 1.14	0.59 ± 0.98	0.351
ETR	12.80 ± 7.40	12.80 ± 15.09	12.68 ± 15.22	0.725
NLNs	13.34 ± 11.28	13.35 ± 11.30	13.34 ± 11.24	0.968
NSR	0.51 ± 1.07	0.52 ± 1.11	0.50 ± 0.95	0.379
NTR	11.07 ± 15.06	11.09 ± 14.98	11.01 ± 15.23	0.829
EPR	8.34 ± 9.55	8.31 ± 9.43	8.40 ± 0.983	0.667
NPR	7.88 ± 9.79	7.86 ± 9.67	7.95 ± 10.07	0.682
Age (years)	** **			0.337
<60	2,816 (29.7)	1,991 (33.0)	825 (29.0)	
≥60	6,671 (70.3)	4,649 (70.0)	2,022 (71.0)	
Sex				0.302
Female	3,498 (36.9)	2,471 (37.2)	1,027 (36.1)	
Male	5,989 (63.1)	4,169 (62.8)	1,820 (63.9)	
Location				0.487
Proximal	3,733 (39.3)	2,589 (39.0)	1,144 (40.2)	
Distal	2,981 (31.4)	2,090 (31.5)	891 (31.3)	
Unknown	2,773 (29.2)	1,961 (29.5)	812 (28.5)	
Histologic type	** **			0.423
Adenocarcinoma	7,227 (76.2)	5,044 (76.0)	2,183 (76.7)	
Mucinous adenocarcinoma	214 (2.3)	158 (2.4)	56 (2.0)	
Signet-ring cell carcinoma	2,046 (21.5)	1,438 (21.7)	608 (21.4)	
Differentiation	** **			0.977
Well	465 (4.9)	324 (4.9)	141 (5.0)	
Moderately	2,757 (29.1)	1,927 (29.0)	830 (29.2)	
Poor/anaplastic	6,265 (66.0)	4,389 (66.1)	1,876 (65.9)	
TNM stage				0.727
I	2,617 (27.6)	1,846 (27.8)	771 (27.1)	
II	2,841 (29.9)	1,976 (29.8)	865 (30.4)	
III	4,029 (42.5)	2,818 (42.4)	1,211 (42.5)	
Clinical cure				
Yes	4,463 (47.1)	3,147 (47.4)	1,316 (46.2)	0.306
No	5,024 (52.9)	3,493 (52.6)	1,513 (53.8)	

* Continuous are shown as the mean ± SD. ELNs, examined lymph nodes; PLNs, positive lymph nodes; NLNs, negative lymph nodes; ETR (ELNs/T-stage) ESR (ELNs/tumor size); NTR (NLNs/T-stage); NSR (NLNs/tumor size); EPR (ELNs/PLNs); NPR (NLNs/PLNs).

### 3.2. Comparison of baseline data before and after matching

**Figure 2:**
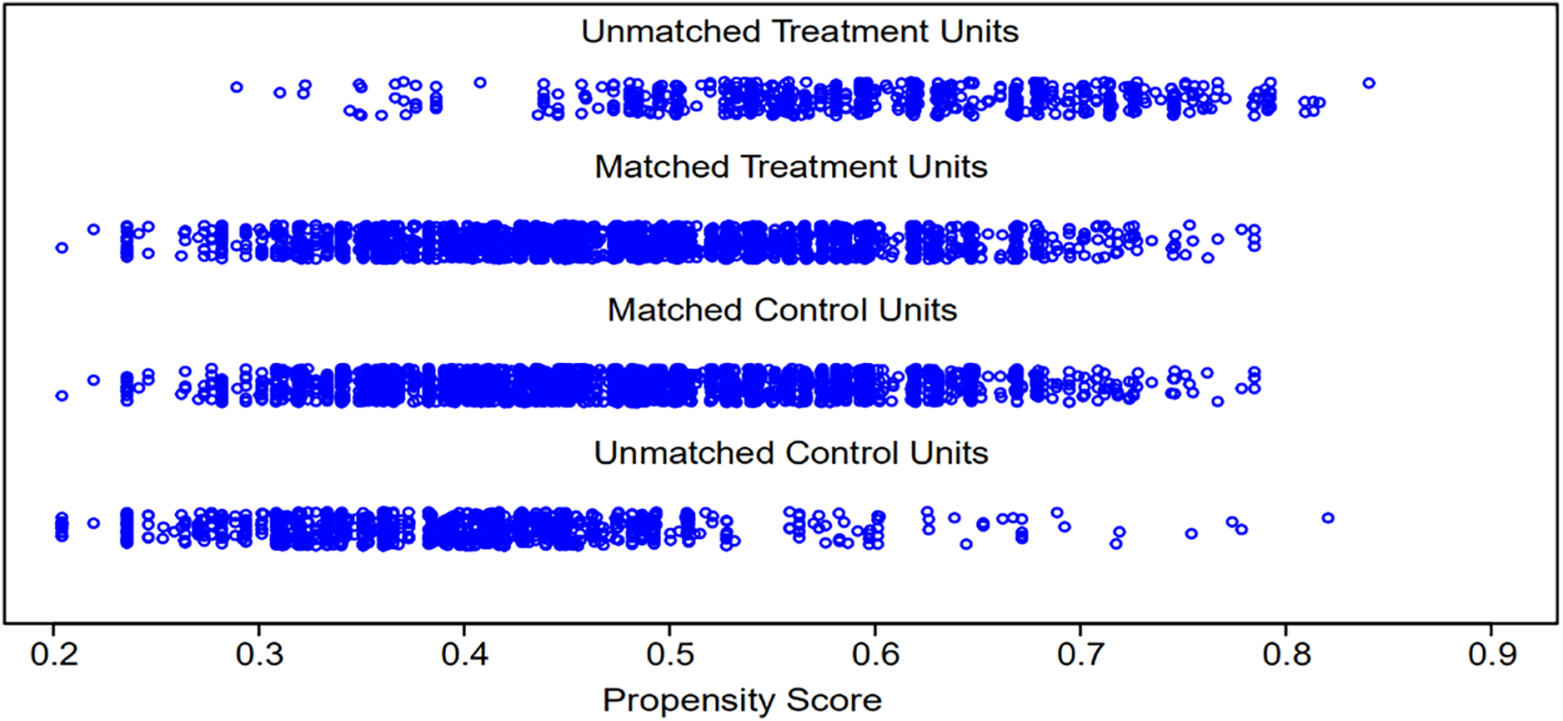
Distribution of propensity scores.

Before matching, in the training cohorts, the numbers of patients older than 60 years, male sex, white race, proximal gastric cancer, signet-ring cell carcinoma, poor or undifferentiated disease and total gastrectomy in the nonclinical cure group were significantly higher than those in the clinical cure group (all *P* < 0.001, Table 2). To avoid selection bias and balance the above significantly different clinicopathological factors (Figure 2), a total of 5,004 cases were chosen according to the chosen 1:1 ratio with a caliper value of 0.005, including 2,502 in each group. After matching, there were no significant differences between the two groups (all P > 0.05, Table 2).

**Table 2:** Distribution profiles of the clinicopathologic factors of the patients in the NCC group and NC group before and after PSM.

Characteristic	Before PSM		*P*	After PSM		*P*
	NCC (*N* = 3,493)	CC (*N* = 3,147)		NCC (*N* = 2,502)	CC (*N* = 2,502)	
Age (years)			<0.001			0.369
<60	1,337 (28.0)	1,014 (32.8)		724 (28.9)	754 (30.1)	
≥60	2,516 (72.0)	2,133 (67.8)		1,778 (71.1)	1,748 (69.9)	
Sex			<0.001			0.977
Female	1,225 (35.1)	1,246 (39.6)		931 (37.2)	933 (37.3)	
Male	2,268 (64.9)	1,901 (60.4)		1,571 (62.8)	1,569 (62.7)	
Race			<0.001			0.235
White	3,430 (68.3)	2,751 (61.6)		1,625 (64.9)	1,659 (66.3)	
Black	683 (13.6)	540 (12.1)		326 (13.0)	282 (11.3)	
Others	908 (18.1)	1,156 (25.9)		549 (21.9)	557 (22.3)	
Unknown	3 (0.1)	16 (0.4)		2 (0.1)	4 (0.2)	
Location			<0.001			0.914
Proximal	1,447 (41.4)	1,142 (36.3)		985 (39.4)	988 (39.5)	
Distal	1,027 (29.4)	1,063 (33.8)		820 (32.8)	807 (32.3)	
Unknown	1,019 (29.2)	942 (29.9)		697 (27.9)	707 (28.3)	
Histologic type			<0.001			0.417
Adenocarcinoma	2,563 (73.4)	2,481 (78.8)		1,936 (77.4)	1,918 (76.7)	
Mucinous	86 (2.5)	72 (2.3)		45 (1.8)	58 (2.3)	
Signet-ring cell	844 (24.2)	594 (18.9)		521 (20.8)	526 (21.0)	
Differentiation			<0.001			0.91
Well	98 (2.8)	226 (7.2)		88 (3.5)	89 (3.6)	
Moderately	851 (24.4)	1,076 (34.2)		753 (30.1)	739 (29.5)	
Poor/anaplastic	2,544 (72.8)	1,845 (58.6)		1,661 (66.4)	1,674 (66.9)	
Surgical method			<0.001			0.989
30	3,105 (61.8)	3,181 (71.3)		1,731 (69.2)	1,736 (69.4)	
40	967 (19.2)	675 (15.1)		403 (16.1)	403 (16.1)	
60	652 (13.0)	455 (10.2)		273 (10.9)	266 (10.6)	
80	300 (6.0)	152 (3.4)		95 (3.8)	97 (3.9)	

PSM, propensity score matching (PSM); CC, clinical cure; NCC, nonclinical cure; 30, represents proximal, distal and half of gastrectomy; 40, represents total gastrectomy; 60, represents adjacent organ resection; 80, represents stomach resection but unclear surgical method.

**Table 3:** The AUC of different markers before and after matching.

Variables	Before PSM		*P* value	After PSM		*P* value
	AUC	95%CI		AUC	95%CI	
ELNs	0.509	0.495–0.523	0.222	0.522	0.506–0.538	0.007
NLNs	0.650	0.637–0.663	<0.001	0.652	0.637–0.667	<0.001
ESR	0.628	0.615–0.642	<0.001	0.622	0.607–0.637	<0.001
ETR	0.697	0.685–0.710	<0.001	0.692	0.678–0.707	<0.001
NSR	0.715	0.703–0.728	<0.001	0.706	0.692–0.720	<0.001
NTR	0.761	0.750–0.773	<0.001	0.751	0.738–0.765	<0.001
EPR	0.753	0.741–0.764	<0.001	0.743	0.730–0.757	<0.001
NPR	0.761	0.749–0.772	<0.001	0.750	0.737–0.764	<0.001

AUC, area under the curve; PSM, propensity score matching (PSM); PLNs, positive lymph nodes; NLNs, negative lymph nodes; ETR (ELNs/T-stage) ESR (ELNs/tumor size); NTR (NLNs/T-stage); NSR (NLNs/tumor size); EPR (ELNs/PLNs); NPR (NLNs/PLNs).

**Table 4:** The predictive value of NTR in the validation cohort.

Group	Predictive cure group	Predictive noncure group	Total
Clinical cure group	879	437	1,316
Nonclinical cure group	493	1,038	1,531
Total	1,372	1,475	

NTR (positive lymph nodes/T-stage).

### 3.3. The predictive value of lymph node-related derived indicators for clinical cure

**Figure 3:**
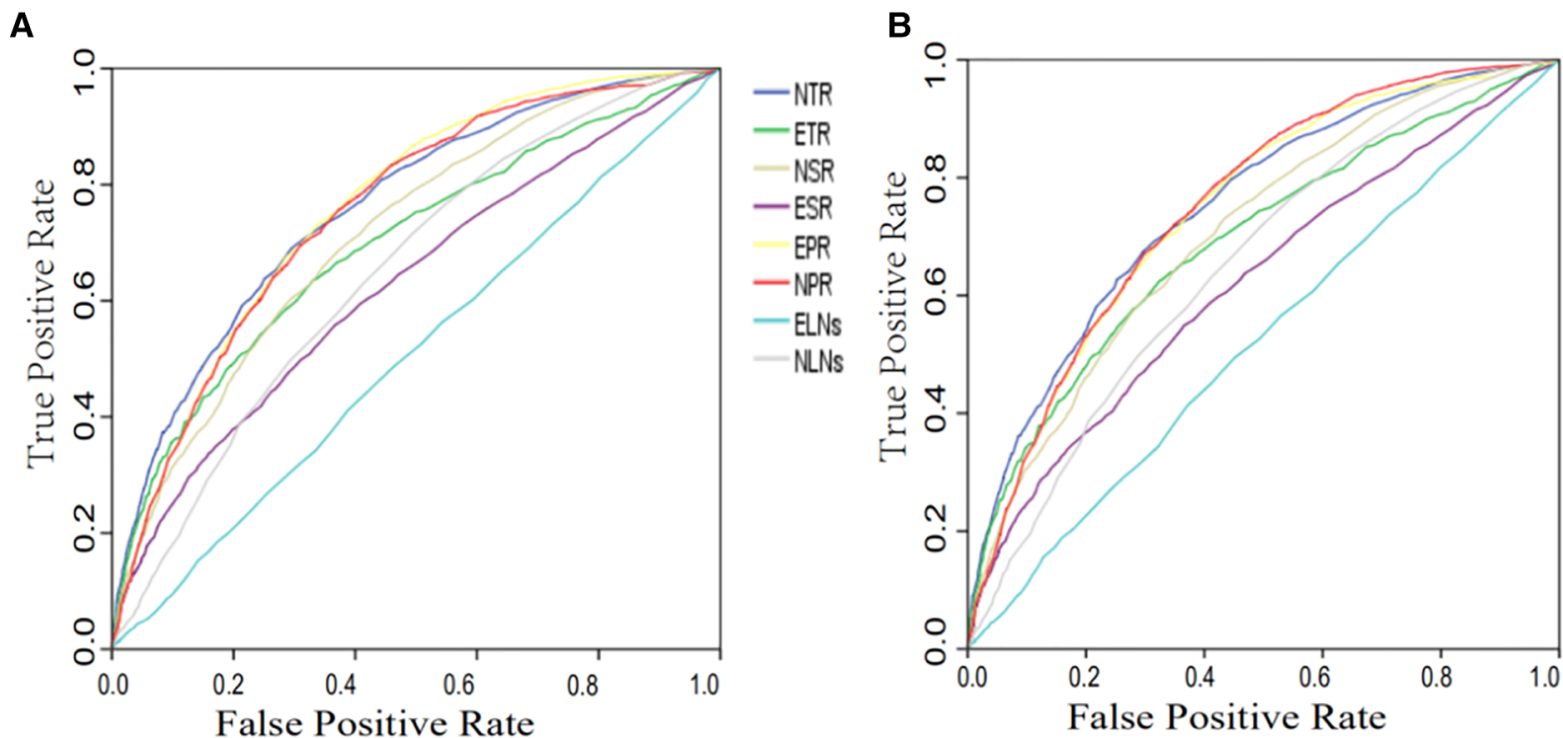
ROC curve of different markers for clinical cure prediction before (**A**) and after (**B**) matching. ELNs, examined lymph nodes; PLNs, positive lymph nodes; NLNs, negative lymph nodes; ETR (ELNs/T-stage) ESR (ELNs/tumor size); NTR (NLNs/T-stage); NSR (NLNs/tumor size); EPR (ELNs/PLNs); NPR (NLNs/PLNs).

A survival time greater than 5 years was used as the gold standard of clinical cure to draw the ROC curve. The ELNs, ETR, ESR, EPR, NLNs, NPR, NTR and NSR were used to construct ROC curves (Figure 3). Before matching, the AUCs of ELNs, NLNs, ESR, ETR, NSR, NTR, EPR and NPR were 0.509, 0.650, 0.628, 0.697, 0.715, 0.761, 0.753 and 0.761, respectively (Table 3). After matching, the AUCs of ELNs, NLNs, ESR, ETR, NSR, NTR, EPR and NPR were 0.522, 0.625, 0.622, 0.692, 0.706, 0.751, 0.743, and 0.750, respectively (Table 3). The predictive ability of ELNs and NLNs was significantly improved after adjusting for T stage, PLNs and tumor size. The predictive efficacy of all markers showed no significant changes before and after matching, and the NTR and NPR had the highest predictive ability. When the cutoff value of NTR was 5.9, there was the highest Youden index (0.378), and the sensitivity and specificity of predictive ability were 67.5% and 70.3%, respectively, in the training cohorts. When the cutoff value of NPR was 4.65, there was the highest Youden index (0.373), and the sensitivity and specificity of predictive ability were 67.0% and 70.3%, respectively, in training cohorts.

### 3.4. Decision curve analysis of ELNs, NLNs and derived markers

**Figure 4:**
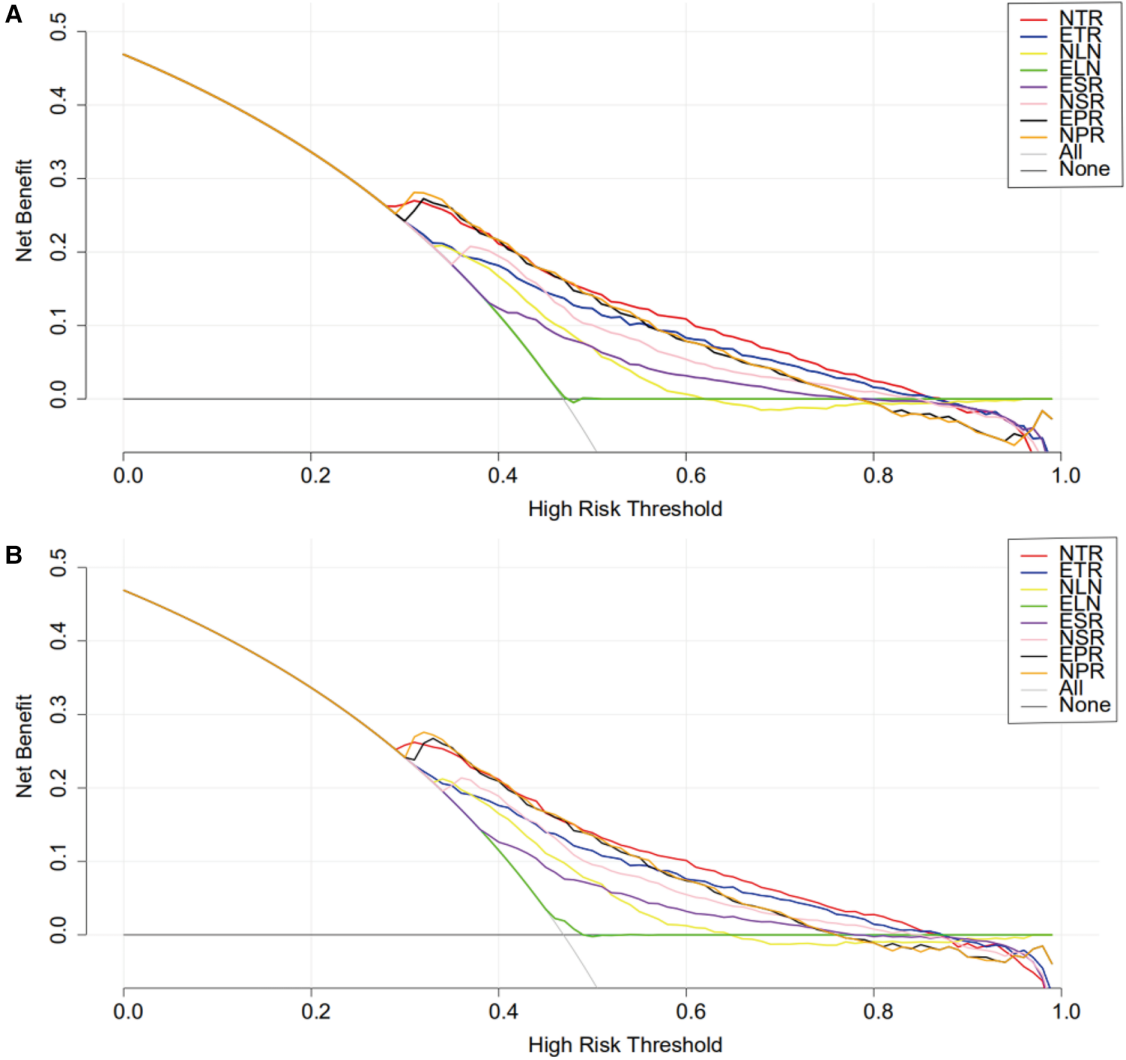
The decision analysis of different indexes before (**A**) and after (**B**) matching. ELNs, examined lymph nodes; PLNs, positive lymph nodes; NLNs, negative lymph nodes; ETR (ELNs/T-stage) ESR (ELNs/tumor size); NTR (NLNs/T-stage); NSR (NLNs/tumor size); EPR (ELNs/PLNs); NPR (NLNs/PLNs).

The advantage of DCA was that the patients’ and decision-makers' preferences were integrated into the analysis to clarify the indexes of clinical benefit (12). To further clarify the clinical benefit and the stability of these markers, the net clinical benefit was drawn as the longitudinal coordinate, the high-risk threshold was drawn as the horizontal coordinate, and the DCA curves of the aforementioned indexes were constructed (Figure 4). The high-risk threshold was set as (0, 1). The net clinical benefits of ESR, ETR, NSR, NTR, EPR, NPR and NLNs were greater than zero, and there was a significant clinical significance. Between the high-risk threshold of 0.3–0.9, the smaller the value of the high-risk threshold is, the higher the net clinical benefit. The overall net clinical benefit of NTR was the highest among all the curves, which means that the clinical benefit of NTR was the largest.

### 3.5. Sensitivity and specificity analysis of NTR in the validation cohort

Combined with the results of ROC and DCA analysis, although NTR and NPR had higher AUCs for clinical cure, the net clinical benefit of NTR was significantly higher than that of NPR. Therefore, we considered NTR to have the best diagnostic efficacy for clinical cures. Based on the optimal cutoff value of NTR obtained in the training cohort (5.9). The validation cohort was grouped into a predictive clinical cure group and a predictive nonclinical cure group. Of 1,316 clinically cured patients, there were 819 positive predictive cases. There were 1,038 negative predictive cases among 1,531 nonclinically cured patients (Table 4). The sensitivity and specificity of NTR for predicting clinical cure outcomes were 66.79% and 67.80%, respectively, similar to those of the training cohort.

### 3.6. Prognostic value of NTR in patients in our department

**Figure 5:**
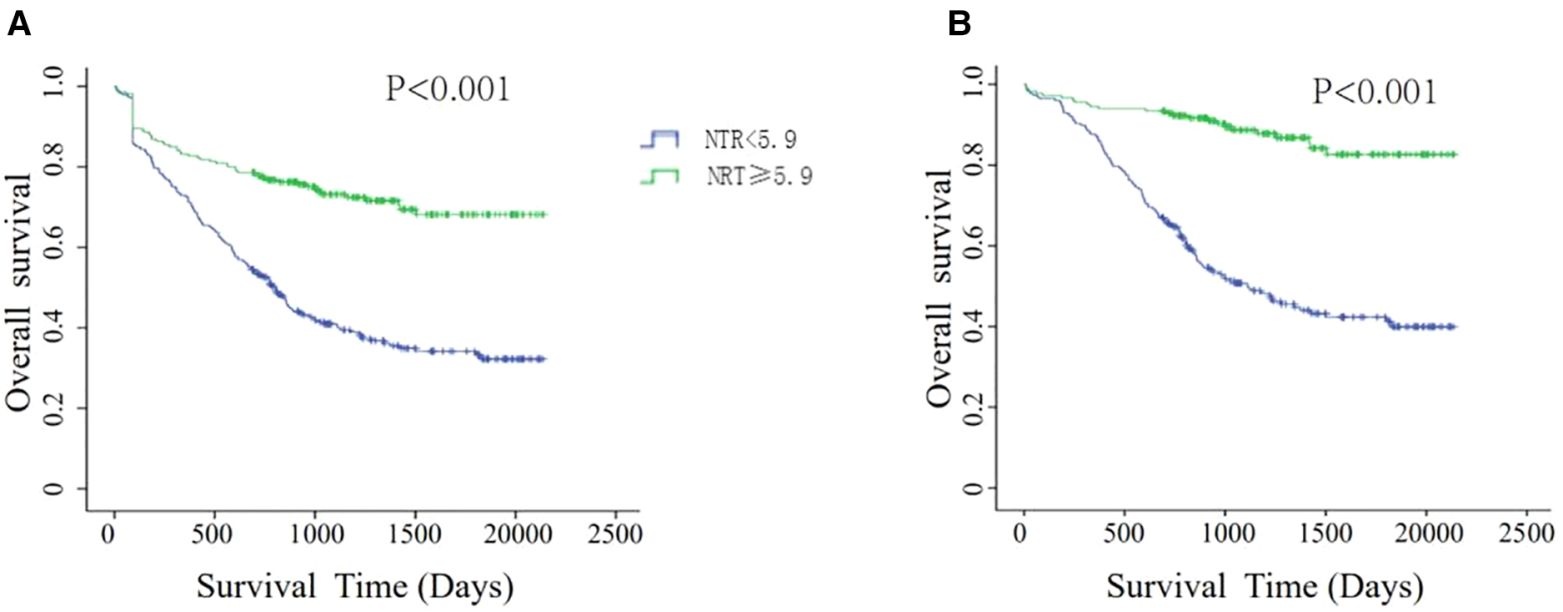
Kaplan–meier survival analyses for overall survival according to NTR scores. (**A**) Patients lost to follow-up were censored at the last known date of contact. (**B**) Patients lost to follow-up were excluded.

Since the number of pathologic stage I-III GC patients who were followed up for more than 5 years was limited in our department, we used survival analysis to validate the clinical value of NTR. The characteristics of this cohort are shown in Supplementary Table S1. Among the 501 patients, 92 patients were lost to follow-up, the loss rate was 18.36%, and the median follow-up time was 872 days (6–2,138 days). According to whether the NTR value was greater than or equal to 5.9, 501 patients were divided into a cured group (*N* = 220) and a noncured group (*N* = 281). As shown in Figure 5, the patients in the cured group had a significantly longer OS than those in the noncured group, regardless of whether the patients lost to follow-up were excluded or their data were censored at the last contact.

To further verify the prognostic value of NTR for patients with different stages, we performed subgroup analysis according to TNM stage. For stages I and III, NTR was not associated with OS. However, for stage II, the cured group had a better OS than the noncured group in any event (Supplementary Figure S2).

## 4. Discussion

In this study, we first used diagnostic experiments and DCA curves to explore the predictors of clinical cure and found that the predictive ability of ELNs and NLNs was significantly improved after adjusting for T stage, PLNs, or tumor size, and the AUC of NTR and NPR had the highest value among these indicators before and after matching. However, the DCA showed that only NTR had the greatest clinical benefit, and NTR also had high specificity and sensitivity in both the training and validation cohorts. When NTR was greater than 5.9, the patients had a better OS in our department. Therefore, we recommend NTR as a predictor of clinical cure.

Recently, many studies on ELNs have mainly focused on precise postoperative staging and prognosis and found that the prognosis of GC was further improved with the increase in the number of ELNs (13–15), which means that the probability of clinical cure would also further increase. One reason for this was that ELNs decreased N stage migration after surgery. Another reason was that perigastric lymph nodes, despite some variations, have a relatively constant number (22–66) and fixed position (16). Therefore, the more ELNs there are, the more thorough dissection and the greater possibility of clinical cure. However, our results show that ELNs had no significant diagnostic value for clinical cure before matching. The AUC of ELNs was 0.509 (*P* = 0.222), which was not statistically significant. The decision curve analysis also suggested that ELNs had no significant benefit for the prediction of clinical outcome. The AUC of ELNs increased to 0.522 (*P* = 0.007) after matching for age, race, histologic type, and other baseline factors, and the DCA suggested a smaller net benefit for ELNs. Therefore, we do not recommend ELNs to predict the clinical cure of GC patients. This may be related to lymph node sorting after surgery. In most cases, ELNs are always less than the total number of retrieved lymph nodes, and ELNs cannot truly reflect the number of retrieved lymph nodes. On the other hand, the more positive lymph nodes in ELNs and the lower number of negative lymph nodes in ELNs indicate a risk of residual cancer. All of these factors may weaken the prediction efficacy of ELNs. Therefore, the predictive value of ELNs should be further studied with standardized lymph node sorting.

NLNs did not include positive lymph nodes. Hence, the higher the value of NLNs is, the more potential metastatic lymph nodes were dissected. NLNs have recently been presented as a research hotspot (13, 17, 18). The increasing number of NLNs not only represents an improvement in the quality of lymphatic dissection but also avoids pathologic stage bias after surgery. Therefore, the number of NLNs has been statistically significant in improving the accuracy of GC patient survival prediction after surgery. Previous studies showed that the residual probabilities of lymph node micrometastases decreased gradually as the number of NLNs increased (19). Our results also showed that the AUC of NLNs was approximately 0.65 before and after matching, which was significantly higher than that of ELNs. DCA showed that the net clinical benefit curve of NLNs was consistently above that of ELNs, further confirming that NLNs were superior to ELNs as a predictor of clinical cure outcomes.

To obtain the best predictor of clinical cure, the ELNs and NLNs were adjusted by T stage, PLNs and tumor size. To ensure the accuracy and generality of the results, patients were randomly divided into training and validation cohorts, and then clinical cure and nonclinical cure groups were matched by PSM for baseline imbalance. This method can simulate randomized controlled trials well and improve the reliability of the results (20). There were significant differences between the clinically cured and noncured groups in age, sex, race, tumor location, surgical methods, and histologic type. Although the ROC and DCA curves all showed that NTR had the highest predictive efficacy and maximum clinical benefit before matching, we could not exclude the influence of these factors (all *P* < 0.001, Table 2). The differences between the two groups in the above factors were significantly reduced after matching (1:1) by PSM (all *P* > 0.05, as shown in Table 2). Surprisingly, we found that the prediction efficiency of ELNs and NLNs was significantly improved after matching with T stage, PLNs and tumor size, which indicates that T stage, PLNs and tumor size were feasible methods to adjust ELNs and NLNs. From the net increase value side, T stage and PNLs were better than tumor size. The ACU suggested that NTR had the highest predictive efficacy for clinical cure (AUC = 0.751), followed by NPR (AUC = 0.750). The NSR (AUC = 0.706), ETR (AUC = 0.692), EPR (AUC = 0.743), and ESR (AUC = 0.622) also had a good predictive ability for clinical cure.

Although the ROC curve can reflect the accuracy of diagnosis through sensitivity, specificity and AUC, it cannot judge the clinical benefit. DCA is used to evaluate the clinical benefit of various indicators and has been widely used to evaluate the clinical efficacy of various models (21, 22). The DCA results are similar to the ROC curve, and NTR had the greatest net clinical benefit. When the risk threshold was 0.3∼0.9, the value of net clinical benefit was greater than 0. The smaller the risk threshold (higher NTR), the higher the clinical benefit. These results showed that patients with more NLNs and earlier T stage had a better prognosis, which was also consistent with the results of previous clinical studies (23–25). However, when the value of the risk threshold was greater than 0.9 or less than 0.3, increased NTR had no additional survival benefit for patients, which meant that extended radical surgery or a low-level increase in NLNs could not improve the clinical cure rate.

When NTR was 5.9, the Youden index of 0.378 was the highest, which meant that 5.9 was the best cutoff value of NTR. In the training group, the sensitivity and specificity of NTR were 67.5% and 70.3%, respectively. The results of the validation group were similar to those of the training group, and their sensitivity and specificity were 66.79% and 67.80%, respectively, indicating that NTR has a high predictive value for clinical cure and that the results are relatively stable. When NTR is less than 5.9, it indicates that the relative lymph node dissection is insufficient, and there is the possibility of micrometastasis in residual LN. Our data also confirm that patients with NTR less than 5.9 exhibited an unfavorable OS, which meant that these patients have a lower possibility of clinical cure, and sufficient attention should be given to postoperative follow-up and treatment strategies. Rather, overtreatment should be avoided for those patients with low risk and NTR greater than 5.9. In clinical application, NTR can be obtained through simple calculation from the postoperative pathological report without additional cost or interpretation of professional background. It is convenient for clinical popularization and application. It is an ideal predictor of long-term clinical outcomes. Its specific prognostic value will be further studied.

However, certain limitations remain to be addressed in the present study. First, the clinical cure group we defined is not a true disease-free state because SEER data do not provide the time of disease-free progression, so there are still a group of patients with tumor survival after 5 years of follow-up, which we cannot distinguish. Second, this study is a retrospective study, which inevitably has a selection bias, such as clinical treatment. Third, although the hazard ratio of the T stage cannot fully reflect the severity of GC, it is closely related to PLNs and many other important tumor characteristics. For clinical application, it is a simple and fast way to predict the severity of cancers, and the prediction efficiency adjusted by T-stage was significantly higher than that adjusted by other indexes, which also proves its superiority. Despite the above shortcomings of our research, we balanced the baseline differences by using a large amount of sample data in the SEER database and propensity score matching. Therefore, the conclusions obtained by using a variety of analysis methods and different datasets are still highly credible.

## 5. Conclusion

NLNs, NTR, NSR, ESR, ETR, NPR and EPR were used as markers for clinical cure. However, NTR was the most effective, and the best cutoff value was 5.9.

## Data Availability

The original contributions presented in the study are included in the article/**Supplementary Material**, further inquiries can be directed to the corresponding author.
