# Supplementary material for: Development and validation of a clinical cure marker based on negative lymph nodes for gastric cancer after gastrectomy
Source: Front Surg. 2023 May 9;10:1016252. doi: 10.3389/fsurg.2023.1016252 (PMC10203492; doi:10.3389/fsurg.2023.1016252)
Supplement: Supplementary file 1 [file Datasheet1.pdf]

Supplementary Material

1 Supplementary Figures

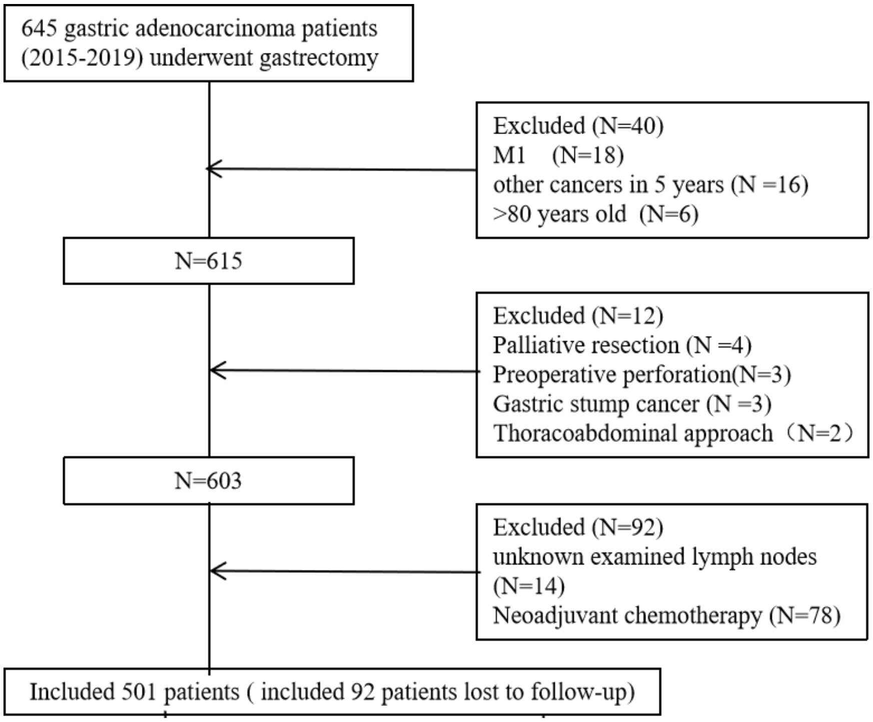

Supplementary Figure 1. Flowchart of patient selection in our department

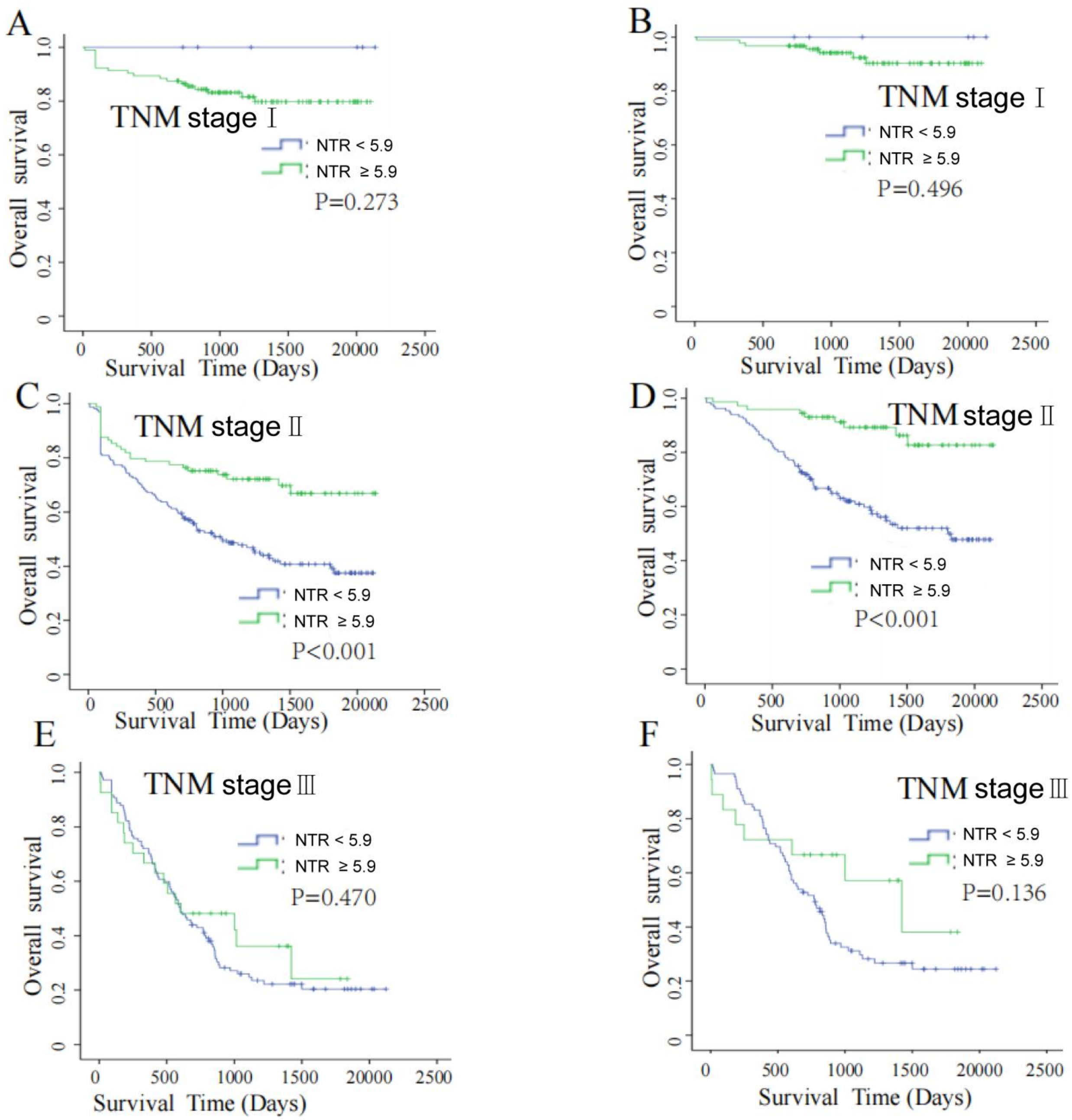

Supplementary Figure 2. Kaplan−Meier survival analyses for overall survival based on NTR scores according to TNM stage. A, C, E: Patients lost to follow-up were censored at the last known date of contact. B, D, F: Patients lost to follow-up were excluded.

2 Supplementary Tables

Supplementary Table 1. Distribution profiles of the clinicopathologic factors of the patients in the nonclinic cure group and clinic cure group

Characteristic	NCC（N=281）	CC（N=220）	NO.	*P*
	No. (%)	No. (%)	（%）	
Age (years)				0.902
＜60	96 (34.2)	74 (33.6)	43.5	
≥60	185 (65.8)	146 (66.4)	44.1	
Sex				0.277
Female	69 (24.56)	45 (20.5)	39.5	
Male	212 (75.44)	175 (79.5)	45.2	
Tumor location				0.001
Upper	124 (44.1)	80 (36.4)	39.2	
Middle	43 (15.3)	25 (11.4)	36.8	
Distal	98 (34.9)	112 (50.9)	53.3	
Total	16 (5.7)	3 (1.3)	15.8	
BMI				0.420
<18.5	38 (13.8)	21 (9.7)	35.6	
18.5-24	167 (60.5)	145 (67.1)	46.5	
>24	71 (25.7)	50 (23.2)	41.3	
Histologic type				0.158
Adenocarcinoma	240 (85.4)	200 (990.9)	45.5	
Mucinous	19 (6.8)	8 (3.6)	29.6	
Signet-ring cell	22 (7.8)	12 (5.5)	35.3	

TNM stage				<0.001
I	6 (2.1)	104 (42.9)	94.5	
II	168 (59.8)	89 (42.9)	34.6	
III	107 (38.1)	27 (14.2)	20.1	
Lost Follow-up				0.577
Yes	54 (19.2)	38(17.3)	41.3	
No	227 (80.8)	182 (82.7)	44.5	

CC, clinical cure; NCC, nonclinical cure;
